# Chronic stress-induced synaptic changes to corticotropin-releasing factor-signaling in the bed nucleus of the stria terminalis

**DOI:** 10.3389/fnbeh.2022.903782

**Published:** 2022-08-02

**Authors:** Isabella Maita, Troy A. Roepke, Benjamin A. Samuels

**Affiliations:** ^1^Samuels Laboratory, Department of Psychology, Behavioral and Systems Neuroscience, Rutgers, The State University of New Jersey, Piscataway, NJ, United States; ^2^Neuroscience Graduate Program, Rutgers, The State University of New Jersey, Piscataway, NJ, United States; ^3^Roepke Laboratory, Department of Animal Sciences, School of Environmental and Biological Sciences, Rutgers, The State University of New Jersey, New Brunswick, NJ, United States

**Keywords:** corticotropin-releasing factor (CRF), bed nucleus of the stria terminalis (BNST), chronic stress, neuroplasticity, CRF1R and CRF2R, PACAP (pituitary adenylate cyclase-activating polypeptide), PAC1

## Abstract

The sexually dimorphic bed nucleus of the stria terminalis (BNST) is comprised of several distinct regions, some of which act as a hub for stress-induced changes in neural circuitry and behavior. In rodents, the anterodorsal BNST is especially affected by chronic exposure to stress, which results in alterations to the corticotropin-releasing factor (CRF)-signaling pathway, including CRF receptors and upstream regulators. Stress increases cellular excitability in BNST CRF+ neurons by potentiating miniature excitatory postsynaptic current (mEPSC) amplitude, altering the resting membrane potential, and diminishing M-currents (a voltage-gated K+ current that stabilizes membrane potential). Rodent anterodorsal and anterolateral BNST neurons are also critical regulators of behavior, including avoidance of aversive contexts and fear learning (especially that of sustained threats). These rodent behaviors are historically associated with anxiety. Furthermore, BNST is implicated in stress-related mood disorders, including anxiety and Post-Traumatic Stress Disorders in humans, and may be linked to sex differences found in mood disorders.

## Introduction

Mood disorders such as anxiety and depression can be triggered or exacerbated by stress, so much research has focused on brain regions that are altered by exposure to chronic or acute stressors. Over the past decade, a region of the extended amygdala, the bed nucleus of the stria terminalis (BNST), has been established as a key player in the stress response. In rodents, the BNST is ventral and anterior to the amygdala proper. Brain anatomy differs in humans, where the BNST is surrounded by the internal capsule, suprascapular pallidal tissue, basal forebrain region, and preoptic area (Avery et al., 2016). The BNST is interconnected with several limbic structures. BNST subnuclei receive inputs from the amygdala, hippocampus, and nucleus accumbens. BNST integrates these inputs and sends projections to the hypothalamus via opposing circuits that tightly regulate the Hypothalamo-Pituitary Adrenal (HPA) axis response (Maita et al., 2021). These structural and functional connections between the BNST and the HPA axis are relatively well-established, suggesting a major role for the BNST in stress-related behavior. Furthermore, BNST sends projections back to limbic areas, and these reciprocal connections play a significant role in learning (Asok et al., 2018) and expressing (Maita et al., 2021) sustained fear states.

Cellular level studies have demonstrated stress-induced synaptic changes in BNST neurons. Neuroplastic changes in the BNST are necessary for fear learning, wherein exposure to sustained or unpredictable aversive stimuli causes changes at the synaptic level (Conrad et al., 2011; Haufler et al., 2013; Goode and Maren, 2017; Goode et al., 2019). Several recent studies have demonstrated synaptic and electrophysiological changes after exposure to chronic stress (Makino et al., 1994; Vyas et al., 2003; Forray and Gysling, 2004; Hammack et al., 2009; McElligott et al., 2010; Conrad et al., 2011; Ventura-Silva et al., 2012; Dabrowska et al., 2013, 2016; Partridge et al., 2016; Snyder et al., 2019; Hu et al., 2020a,b). Much of these changes occur within corticotropin-releasing factor (CRF)-expressing neurons. This review will give a brief overview of the stress-related neural circuitry and microcircuitry of the BNST, and then will detail studies investigating synaptic changes in CRF+ neurons in the BNST during fear learning and after exposure to stress. This link between stress and neuroplastic changes to CRF functioning in BNST is important for understanding stress-related disorders in humans, including mood disorders such as anxiety, post-traumatic stress disorder (PTSD), and major depressive disoder, as well as substance abuse disorders. Finally, sex differences in CRF-signaling in the BNST observed in both rodents and humans may hint at mechanisms underlying sex differences in human mood disorder.

### Corticotropin-releasing factor and the stress response

Corticotropin-releasing factor (CRF), a 41 amino-acid peptide neuromodulator (Rivier et al., 1983), is a hypophysiotropic hormone that is a major component of the stress response. At the behavioral level, CRF administered into the Central Nervous System (CNS) of rodents via intracerebroventricular (ICV) infusion increases avoidance of innately aversive contexts, such as bright lights and open spaces in the open field (OF) test (Sutton et al., 1982). These avoidance behaviors in rodents are decreased by anxiolytic drugs, and thus are historically associated with anxiety. Importantly, exposure to stress similarly causes increased avoidance of aversive contexts.

CRF-expressing neurons densely populate the paraventricular nucleus (PVN) of the hypothalamus (De Souza, 1987). When released from the PVN, CRF (also referred to as CRH) stimulates the release of adrenocorticotrophic hormone (ACTH) from the anterior pituitary, ultimately increasing systemic corticosteroids, glucocorticoids, and mineralocorticoids, and activating the stress response, a process thoroughly reviewed by Owens and Nemeroff (1991). CRF also modulates the HPA axis indirectly, via actions in areas such as the amygdala and BNST.

Several components of the CRF-signaling pathway are implicated in the stress-response. CRF can bind at two G-protein coupled receptor subtypes, CRFR1 and CRFR2, which are dispersed throughout the brain, including the BNST, hippocampus, and anterior and lateral hypothalamus (Chalmers et al., 1995). In the PVN, CRFR1 expression is surprisingly low (Potter et al., 1994) to moderate, while CRFR2 is moderately expressed in the medial PVN (Chalmers et al., 1995). CRFR1-expressing neurons in the PVN receive inputs from neighboring hypothalamic interneurons, and, to a lesser extent, the BNST, lateral septum, preoptic area, and supraoptic nucleus (Jiang et al., 2018). Importantly, experiments measuring CRFR expression rely on *in situ* hybridization, which measures somatic mRNA and not synaptic protein expression, where CRFRs typically localize (Swinny et al., 2003; Liu et al., 2004). The CRF signaling pathway is modulated by upstream regulator pituitary adenylate cyclase activating polypeptide (PACAP) and its receptor PAC1, which regulates avoidant behavior (Hammack et al., 2009; Kocho-Schellenberg et al., 2014). Striatal-enriched protein tyrosine phosphatase (STEP) is a known upstream inhibitor of CRF, and is also implicated in the stress-response (Hammack et al., 2009; Hu et al., 2020a,b).

As reviewed by Shekhar et al. (2005), the basolateral amygdala is known to exhibit increased sensitivity after stress, likely due to CRF-driven long term potentiation, promoting increased avoidance responses. Several recent studies have suggested the extended amygdala, especially the BNST, also plays an integral role in mediating the maladaptive effects of chronic stress (Lebow and Chen, 2016). CRF-producing neurons are enriched in the anterolateral and anterodorsal subnuclei of BNST, and there is strong evidence that neuroplasticity in BNST CRF neurons drive the stress-response (Critchlow et al., 1963; Karandrea et al., 2000; Bangasser et al., 2005; Perry et al., 2005; Gourley and Taylor, 2009; Hammack et al., 2009; Dabrowska et al., 2013; Marcinkiewcz et al., 2016; Partridge et al., 2016).

### Corticotropin-releasing factor in the bed nucleus of the stria terminalis

#### Overview

The BNST is innervated by limbic structures including the amygdala, hippocampus, nucleus accumbens, and medial prefrontal cortex, as well as the dorsal raphe and ventral tegmental area (VTA). These inputs synapse onto the highly complex and heterogenous system of interneurons within the BNST. BNST subnuclei consist of varying cell types with diverse molecular signals, electrophysiological responses, receptor expression, local connectivity, and distal connectivity (Gungor and Pare, 2016). The majority of BNST neurons are GABAergic, and either inhibit local BNST projections or project to other areas, including the hypothalamus (Dabrowska et al., 2016; Giardino et al., 2018), dorsal raphe nucleus, VTA (Dabrowska et al., 2016), and nucleus accumbens (Dong et al., 2001; Taha and Fields, 2005). Given these unique anatomical connections, Lebow and Chen (2016) proposed a “valence surveillance” hypothesis where the BNST responds to external stimuli with either positive or negative valence. The BNST then integrates this information to regulate avoidance behavior via hypothalamic projections and reward-driven behavior through projections to the VTA and nucleus accumbens. As reviewed by Maita et al. (2021), subnuclei from both the anterior and posterior regions of the BNST strongly innervate the hypothalamus, synapsing mainly at two hypothalamic regions that are known to regulate homeostatic functions, social behaviors, and the stress response: the lateral hypothalamus (LH) and the PVN.

The BNST can be divided into anterior and posterior regions, which can be further split into 12–18 subnuclei according to anatomical divisions in rats. The anatomy of the mouse BNST subnuclei are debated because they are less distinct than what is observed in rats. This review utilizes anatomical characterizations from Dong and Swanson (Dong et al., 2001; Dong and Swanson, 2003, 2004a,2004b, 2006a,2006b,2006c). The posterior BNST is made up of the principal, transverse, and intrafascicular subnuclei, and is known to regulate mating and reproductive behaviors, as well as homeostatic functions such as osmotic regulation and thirst. The anterior region includes the oval, anteromedial, dorsomedial, magnocellular, anterolateral, anteroventral/ventral, rhomboid, juxtacapsular, and fusiform nuclei (Allen Mouse Brain Atlas, 2004). The anterior BNST is critical for the behavioral consequences of chronic stress, and is highly connected to the HPA axis (Gungor and Pare, 2016).

The anterior medial group of the BNST includes four subnuclei that are involved in the stress response and fear learning. These subnuclei include the **anteromedial nucleus**, which receives most of the inputs to the BNST, notably, glutamatergic inputs from the basomedial amygdala, glutamatergic inputs from the ventral subiculum implicated in anxiolysis (Glangetas et al., 2017), and GABAergic inputs from the medial amygdala implicated in fear conditioning (Haufler et al., 2013). The **dorsomedial** and **magnocellular nuclei** play a major role in hypothalamus inhibition, via GABAergic outputs to the PVN (Dong and Swanson, 2006b). Lastly, a population of glutamatergic neurons in the **anteroventral nucleus** have an excitatory effect on PVN (Gungor and Pare, 2016), and may be important for the valence surveillance role of the BNST (Lebow and Chen, 2016). However, unlike the anterior BNST, there is no evidence that CRF signaling regulates the anterior medial group.

#### Corticotropin-releasing factor-expressing subnuclei in the anterior bed nucleus of the stria terminalis

**FIGURE 1:**
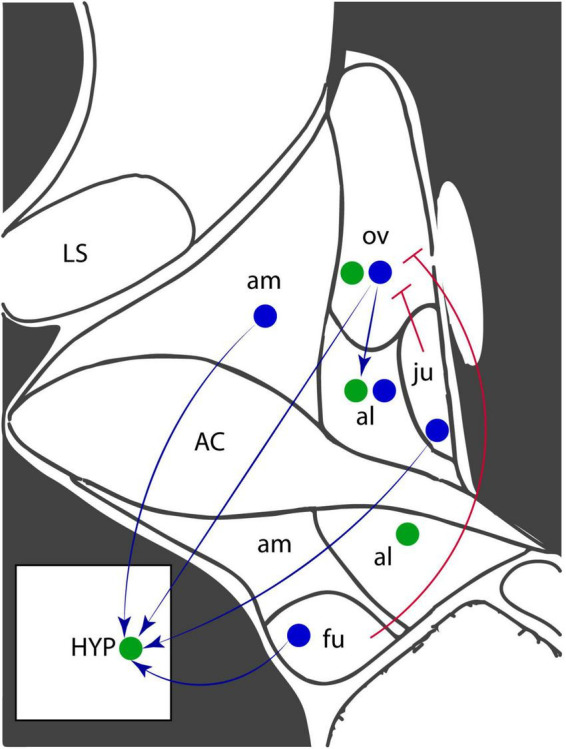
Corticotropin-releasing factor (CRF) and receptor expression in the anterodorsal region of the BNST. Coronal diagram of the mouse anterior BNST, based on the Allen Brain Atlas. Blue circles represent CRF-expressing neurons and green circles represent CRF receptor-expressing neurons. Arrows indicate projections while blunted arrowheads indicate inhibitory projections. AC, anterior commissure; al, anterolateral nucleus; am, anteromedial nucleus; fu, fusiform nucleus; HYP, hypothalamus; LS, lateral septum; ju, juxtacapsular nucleus; ov, oval nucleus.

CRF-signaling in the anterior BNST, especially the anterodorsal and anterolateral regions, is a well-established component of the stress response (Lebow and Chen, 2016). CRF-expressing neurons project within the BNST, creating microcircuit connections between subnuclei, and to external projections, including the hypothalamus (Figure 1). Both CRFR1 and CRFR2 receptors are highly expressed throughout the BNST (Chalmers et al., 1995). The **oval nucleus** (ovBNST) is an anterodorsally-located subnucleus populated by a majority-population of CRF-expressing GABAergic neurons (Forray and Gysling, 2004). The ovBNST CRF neurons project to and inhibit areas involved in both avoidance and reward behaviors, including the LH (Giardino et al., 2018), PVN, dorsal raphe nucleus (Dabrowska et al., 2016), VTA (Dabrowska et al., 2016), and nucleus accumbens (Dong et al., 2001; Taha and Fields, 2005). Dopamine receptor-expressing neurons in the ovBNST, some of which co-express CRF (Giardino et al., 2018), are activated by dopaminergic inputs from the VTA and dorsal raphe nucleus (Park et al., 2013). The ovBNST has been more highly researched relative to other subnuclei, likely due to its homogeneity and relatively straightforward microcircuitry. The **rhomboid** and **juxtacapsular** nuclei are also anterodorsal nuclei populated by GABAergic interneurons that inhibit CRF+ neurons in the ovBNST and amygdala (Dong and Swanson, 2003). The juxtacapsular nucleus also contains GABAergic CRF+ neurons that project to the LH and to CRF+ ovBNST neurons (Francesconi et al., 2009; Giardino et al., 2018).

Several key limbic areas also synapse onto the **anterolateral nucleus,** including the ventral subiculum, dorsal raphe, and amygdala (Gungor and Pare, 2016). CRF+ ovBNST neurons synapse onto CRFR1-expressing anterolateral BNST neurons (Dong and Swanson, 2004a). Opposing circuitry in the anterolateral BNST regulates the stress response (Gungor and Pare, 2016). While most BNST subnuclei are thought to promote avoidance, stimulation of the anterolateral BNST decreases corticosterone release and inhibition increases behavioral fear responses, suggesting a role in inhibiting HPA axis responses (Gungor and Pare, 2016). The CRF-expressing neurons in the ovBNST and anterolateral BNST, especially, are critical components of the rodent response to sustained stress during both chronic stress and fear learning paradigms.

Some anteroventral neurons are also involved in the stress response and fear learning. These neurons express serotonin receptors and receive inputs from the dorsal raphe nucleus (Marcinkiewcz et al., 2016). Ventrally-located **fusiform nucleus** neurons express CRF, releasing it onto the CeA, PVN, nucleus accumbens, and PAG (Dong et al., 2001). Unlike the glutamatergic inputs to the ovBNST, CRF neurons in the fusiform nucleus receive mainly noradrenergic fibers from the brain stem and locus coeruleus (Dong et al., 2001). Microcircuitry between BNST subnuclei is complex. Dense projections from the ovBNST innervate the fusiform nucleus, and less dense projections innervate the anterolateral BNST, and rhomboid nucleus. The fusiform nucleus also sends dense projections, which innervate the dorsomedial BNST and anterolateral BNST (Dong et al., 2001). CRF-expressing CeA neurons synapse throughout the ventral subnuclei of the BNST, releasing CRF onto both CRF- and CRF+ BNST neurons (Partridge et al., 2016).

The BNST is highly heterogeneous and is modulated by many other neurotransmitters and neuromodulators beyond CRF, some of which affect CRF signaling and the stress response (Kash et al., 2015). For example, opiate-like neuropeptide Nociceptin/Orphanin FQ has anxiolytic properties (Rodi et al., 2008), but BNST neurons expressing its precursor, Prepronociceptin (Pnoc), are mainly GABAergic, and promote arousal and avoidance behaviors. There is little overlap between CRF and Pnoc expression in the BNST (Rodriguez-Romaguera et al., 2020). Dynorphin, a second neuropeptide with opioid action, may interact synergistically with CRF signaling to promote avoidance behavior (Bruchas et al., 2009). A third neuromodulator, calcitonin gene-related peptide (CGRP) may also act synergistically with CRF in the anterolateral BNST; CGRP may increase CRF+ anterolateral BNST neuron activity and CRFR1 inactivation reduces acoustic startle potentiation caused by CGRP infusion (Sink et al., 2013a; Gungor and Pare, 2014). By contrast, Neuropeptide Y may oppose CRF function in the BNST (Kash and Winder, 2006). Further investigation is required to understand how CRF signaling overlaps and interacts with other neuromodulator signaling pathways in BNST to mediate the stress response.

## Neuroplasticity in the corticotropin-releasing factor-signaling pathway

Activation of the whole BNST increases avoidance behavior (Dong et al., 2001) while lesions decrease stress-induced avoidance behavior (Hammack et al., 2004). CRF infusion directly into the BNST also increases avoidance behaviors (Sahuque et al., 2006). More recent studies using circuit-level approaches to modulate specific BNST cell populations show that most BNST neurons, including the CRF+ population, increase avoidance behaviors when activated (Kim et al., 2013; Gungor and Pare, 2016; Marcinkiewcz et al., 2016; Giardino et al., 2018; Giardino et al., 2018; Yamamoto et al., 2018; Hu et al., 2020a). Optogenetic activation of neurons expressing dopamine receptor 1 (Drd1), which is reported to colocalize with CRF in ovBNST neurons, increases avoidance in open field and elevated plus maze (EPM) tests (Kim et al., 2013; Hu et al., 2020a) and increases respiration, a physiological sign of stress (Kim et al., 2013). By contrast, inhibition of these ovBNST neurons is rewarding, and decreases avoidance and respiratory rate (Kim et al., 2013). Another population of CRF-expressing BNST neurons project to the LH. Activation of these neurons is also aversive (Giardino et al., 2018). These data suggest that a major mechanism underlying maladaptive behavioral stress responses may be modulation of BNST CRF + neurons. Much work has attempted to address this question through a variety of different behavioral outcomes, stress procedures, and cellular approaches. The following sections will detail these studies. Unfortunately, many of these studies only used male rodents (unless otherwise specified). As described later in the review, several components of the CRF signaling pathway may differ between males and females.

### Sustained fear learning recruits the bed nucleus of the stria terminalis

In addition to avoidance behaviors, stress exposure alters the fear response through BNST CRF+ neurons. During fear learning, acute stressors, such as foot-shock, are associated with conditioned stimuli, such as a cue or context. The rodent central amygdala (CeA) is activated by cues that predictably and immediately precede the stressor, such as a tone that plays under 10 s prior to a foot-shock. These cues are said to elicit short-term or phasic fear. By contrast, the rodent BNST is activated by unpredictable cues and sustained cues that are said to elicit long-term or sustained fear. Sustained cues are those that precede a temporally distant stimulus, such as a tone that warns of a foot-shock that occurs over 30 s later. Unpredictable cues inconsistently precede aversive stimuli, such as a tone that only sometimes precedes shock (Shackman and Fox, 2016; Goode and Maren, 2017; Goode et al., 2019).

Lesions to the BNST reduce expression of contextual fear learning in both male and female rats (Urien et al., 2021). However, the story becomes more complex when looking at specific subnuclei. Following fear conditioning, both associated context-exposure and cue-exposure elicit opposite changes in responsivity from BNST neurons: firing increases in anteromedial BNST neurons and decreases in anterolateral BNST neurons (Haufler et al., 2013).

Interestingly, in humans, unpredictable long-duration threat (Alvarez et al., 2011) and late anticipatory phases prior to threat (McMenamin et al., 2014) also induce BNST activity. The disparate functional roles assigned to the rodent CeA and BNST- phasic and sustained fear, respectively- may not apply to primates. Short term threat elicits BNST activity in humans (Choi et al., 2012; Klumpers et al., 2015) and non-human primates (Shackman and Fox, 2016) and the CeA may also be activated by sustained fear (Shackman and Fox, 2016).

In rodents, CRF is at least partially responsible for BNST regulation of fear learning. Male mice exposed to contextual fear conditioning exhibited higher CRF expression in anterolateral BNST neurons (Urien et al., 2021). Overexpression of CRF in the BNST (Sink et al., 2013b) and activation of a dorsal raphe nucleus projections onto CRF+ BNST neurons improve cued and contextual fear recall (Marcinkiewcz et al., 2016). Furthermore, inhibition of CeA projections onto CRF+ dorsolateral BNST neurons disrupts sustained contextual fear learning (Asok et al., 2018). These effects are projection-specific: depletion of CRF from VTA-projecting neurons, including those in the anterior BNST, increases freezing during cued fear learning (Dedic et al., 2018). However, VTA CRF depletion may cause increased sensitivity to stress because subjects also exhibited higher freezing to an unpaired tone (Dedic et al., 2018). Acute stressors improve connectivity in fear-learning pathways, permitting improved acquisition of cue-shock associations (Goode and Maren, 2017; Goode et al., 2019). This effect is BNST-dependent, as this enhancement is blocked when BNST is inhibited during training (Bangasser et al., 2005). There is also evidence of sex differences in neuroplastic changes to the BNST during fear learning. Male rats, but not females, display upregulated immediate early gene, Arc, in the anterolateral BNST after expression of contextual fear conditioning (Urien et al., 2021). Future studies must consider sex differences as a factor when investigating neuroplasticity after fear conditioning.

Both acute and chronic stress paradigms used in rodents can alter BNST neuroplasticity. However, acute and chronic stress paradigms have significantly different translational validity. In humans, repeated, chronic stress exposures are more likely to precipitate mood disorders such as anxiety and major depressive disorder than a single, acute stress exposure. In rodents, chronic stress paradigms expose rodents to stressful experiences over periods of several days to weeks. By contrast, acute stressors are strongly aversive stimuli that, after a limited number of exposures, produces sensitization to similar or weaker stimuli.

Acute and chronic stress exposures can cause different neural responses. For example, acute stress enhances fear learning, but exposure to chronic stress appears to decrease synaptic strength in the BNST and impair fear learning (Conrad et al., 2011). Given the better translational validity for understanding the etiology of mood disorders such as anxiety and major depressive disorder, we will focus specifically on the effects of chronic stress in this review. Many chronic stress paradigms can induce maladaptive behavioral states in rodents. However, several of these chronic stress paradigms were designed in, optimized for, and only are effective in inbred strains of male rodents. Given sex differences in CRF signaling in the BNST, the next section will explain several historically used chronic stress paradigms and then highlight more recently developed paradigms that permit the study of stress in both male and female rodents.

### Chronic stress paradigms

Several different chronic stress paradigms (Table 1) are used in rodents. These paradigms exploit a variety of innately stressful conditions to elicit behavioral and neuroendocrinal stress-responses, including increased avoidance and HPA axis activation. One often used approach is to mimic chronic stress via a pharmacological intervention, such as chronic administration of corticosterone, the primary adrenal corticosteroid produced in rodents. Chronic corticosterone administration via subcutaneous injection, slow-release pellet implantation, (Makino et al., 1994) or drinking water (David et al., 2009; Gourley and Taylor, 2009; Dieterich et al., 2019) results in increased avoidance behaviors and increased CRF levels. However, corticosterone administration only causes these behavioral effects in male rodents (Mekiri et al., 2017; Yohn et al., 2019).

**TABLE 1:** Behavioral paradigms commonly used to induce stress-response in rodents.

Paradigm	Summary	Stressor type	Sex	Total duration	Daily duration
Chronic restraint stress	Daily episodes of restricted movement	Physical	M+F	5–30 days	5–180 min
Chronic corticosterone	Continuous administration of corticosterone dissolved in drinking water	Pharmacological	M+F	21–35 days	O/N
Chronic variable mild stress	Daily exposure to randomized micro- stressors, i.e., predator odor, restraint stress, poor housing	Varies	M+F	14–60 days	3–4 h
Chronic non- discriminatory social defeat stress	Daily physical bouts with aggressor mouse and co-habituation	Social, physical, emotional	M+F	10 days	5–10 min + O/N
Social instability stress	Unstable social hierarchy through cage-mate changes	Social	M+F	30 days	O/N
Chronic social defeat stress	Daily physical bouts with aggressor mouse and co-habituation	Social, physical	M	10 days	5–10 min + O/N
Vicarious social defeat stress	Female mice witness social defeat of male mice	Social, emotional	F	10 days	5–10 min

Paradigms vary by methodology, type of stressor, duration and in which sex the paradigm effectively elicits a stress-response. More invasive techniques described in the main text. F, female; M, male; O/N, overnight.

Another paradigm is chronic immobilization or restraint stress (RS). Chronic RS is a simple, easily replicated physical stress paradigm, wherein rodents are subject to daily episodes of restricted movement. The paradigm is easily adjusted; episode duration typically varies between 15 and 180 min and the number of exposures ranges between 5 and 30 days (Pare and Glavin, 1986). Other widely used paradigms, such as chronic variable mild stress (CVMS) and unpredictable chronic mild stress (UCMS) use exposure to changing daily stressors, so subjects do not habituate to any one stimulus. Examples include social stressors such as singly housing rodents or introducing unfamiliar cage-mates to disrupt the existing hierarchy of the cage, and physical stressors such as altering light/dark schedules, restricting food or water, or creating aversive housing conditions by wetting the bedding or tilting the cage. Repeated cage-changes, which removes familiar odors and nests, is also a commonly used stressor as are exposure to predator sounds or odors (Hill et al., 2012; Monteiro et al., 2015; Willner, 2017; Antoniuk et al., 2019). The CVMS and UCMS paradigms utilize a varying schedule of these short-term stressors, typically one or two different stressors per day over the course of weeks. Other variations of these paradigms also exist that use the same stressors repeatedly and/or over long periods of time.

Early life stress paradigms also use a variety of stressors on rodents within their first weeks, to reliably produce a stressed phenotype later in life. Stressors include early weaning, maternal separation, handling, and impoverished housing environments (Schmidt et al., 2011). Early life stress, as well as forced swim stress, during which rodents undergo daily placement in an inescapable swimming chamber, more reliably alter motivated or reward-driven behavior, rather than avoidance behaviors (Yankelevitch-Yahav et al., 2015). Importantly, CVMS, UCMS, early life stress, and forced swim stress can be used to produce a stress response in both male and female rodents.

Another behavioral chronic stress paradigm, Chronic Social Defeat Stress (CSDS), uses social and physical stressors by instigating daily physical defeat bouts by larger, aggressive mice, and then co-housing mice with their aggressor, separated by a divider (Golden et al., 2011). CSDS results in both susceptible and resilient phenotypes in inbred C57BL6/J male mice, which may in part represent the spectrum of human responses to stressors. After exposure to stress, susceptible male mice exhibit increased social avoidance, spending less time with a novel rodent in the Social Interaction Test (SIT) compared to control counterparts. Resilient subjects have undergone CSDS but behave comparably to controls in the SIT and other tests of avoidance and do not exhibit stress-induced hippocampal neuroplasticity seen in susceptible subjects (Lee et al., 2021). Future experiments should consider whether neuroplasticity in CRF+ BNST neurons affects stress resilience after social stress.

CSDS works well in male rodents, but aggressive males will more often display mounting behaviors when presented with a female intruder. Female-female aggression can be elicited via ovariectomy of female mice (DeBold and Miczek, 1984). Aggressor male mice can also be provoked to attack females via chemogenetic activation of the ventral medial hypothalamus (Takahashi et al., 2017) or application of male urine to the anogenital region (Harris et al., 2018). Most recently, a variant of CSDS called Chronic Non-discriminatory Social Defeat Stress (CNSDS) was developed to simultaneously perform chronic stress experiments in both male and female rodents. In CNSDS, aggressor mice are driven to attack both sexes when both a male and a female mouse are simultaneously placed into their home cage (Yohn et al., 2019). Importantly, CNSDS results in susceptible and resilient phenotypes in both sexes, and increases avoidance while decreasing reward behaviors (Yohn et al., 2019; Dieterich et al., 2021).

Another paradigm that is effective in both sexes, Social Instability Stress, elicits chronic stress by creating an unpredictable social environment via repeated changes to the social hierarchy, housing unfamiliar mice together (Schmidt et al., 2007, 2010). Another paradigm is Vicarious Defeat Stress, where mice can be exposed to emotional and social, but not physical, stressors by observing a male undergoing CSDS and then being co-housed with the observed aggressor, but separated by a clear, perforated divider (Sial et al., 2016).

### Stress-induced neuroplasticity in the bed nucleus of the stria terminalis

**FIGURE 2:**
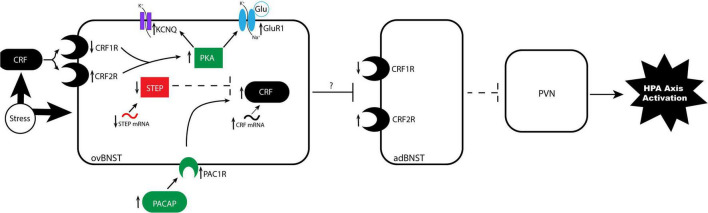
Proposed mechanism of stress-induced neuroplasticity in the anterodorsolateral BNST. In the stressed brain, alterations to CRF G-protein coupled receptors alter sensitivity to CRF binding in undetermined ways. BNST CRFR1 expression is reduced and CRFR2 expression is increased, resulting in altered sensitivity to CRF binding from external projections, such as the CeA. Binding triggers a signaling cascade resulting in increased PKA, which promotes phosphorylation of glutamate receptor GluR1 and phosphorylation of the KCNQ channel. These parallel phosphorylation pathways increase excitability in the ovBNST by increasing mEPSCs and resting membrane potential, respectively. A second pathway regulates CRF signaling, wherein stress increases CRF mRNA, increases upstream regulator PACAP and its receptor PAC1, and decreases upstream inhibitor STEP mRNA and protein production. Increased excitability in ovBNST likely increases GABAergic inhibition of anterodorsal BNST subnuclei that project to the hypothalamus, disinhibiting the PVN. The PVN then releases increased levels of CRF to the anterior pituitary, which activates the stress response in the body. adBNST, anterodorsal BNST; CRF, corticotropin-releasing factor; CRFR1, CRF receptor 1; CRFR2, CRF receptor 2; GluR1, glutamate receptor 1; ovBNST, oval nucleus of the BNST; PACAP, pituitary adenylate cyclase activating polypeptide; PAC1, PACAP receptor 1; PKA, protein kinase A; PVN, paraventricular nucleus of the hypothalamus; STEP, striatal-enriched protein tyrosine phosphatase.

Several of the chronic stress paradigms described above lead to long-lasting behavioral changes in part due to stress-induced alterations to BNST neurons. Chronic RS leads to neuroplastic changes, including increased BNST volume and dendritic arborization, that facilitate avoidance behavior in male rats (Vyas et al., 2003). Different chronic stress paradigms can also cause upregulation of glutamate (Dabrowska et al., 2013; Hu et al., 2020b), GABA (Ventura-Silva et al., 2012), and NMDA (Ventura-Silva et al., 2012; Dabrowska et al., 2013) receptors in the BNST, altering responses to both excitatory and inhibitory inputs (Ventura-Silva et al., 2012). Both chronic social isolation and chronic corticosterone administration induce long term depression (LTD) in glutamatergic anterolateral BNST neurons and blunt long term potentiation (LTP) in anterodorsolateral BNST neurons (Conrad et al., 2011). Therefore, chronic stress exposure may activate a positive feedback mechanism by reducing activity in the anterolateral and anterodorsolateral BNST, thereby disinhibiting CRF release from the PVN, and ultimately decreasing corticosterone release from the anterior pituitary (Gungor and Pare, 2016). However, electrophysiological responses to chronic stressors are heterogenous; in lateral regions of the BNST, stress differentially dysregulates norepinephrine, acetylcholine, and glutamate-facilitated LTD (McElligott et al., 2010). Importantly, chronic stressors induce LTP in CRF+ neurons, which act as interneurons that disinhibit the hypothalamus by increasing inhibition of other GABAergic inputs to the hypothalamus (Gungor and Pare, 2016; Figure 2).

Different chronic stress paradigms and early-life stress can lead to long-lasting increases of CRF and complex alterations in CRF signaling components in BNST. Corticosterone administration increases CRF mRNA in the dorsolateral and ventral BNST (Makino et al., 1994), while CVMS and early life stress result in increased CRF signaling in the BNST of male mice (Forray and Gysling, 2004; Hu et al., 2020a,b). CRF binds at two receptor subtypes, CRFR1 and CRFR2, which are Gs-protein coupled membrane receptors. Using *in situ* hybridization, Ventura-Silva et al. (2012) found that UCMS decreases CRFR1 mRNA in the dorsomedial and fusiform nuclei of the BNST, but increases CRFR2 mRNA in the principal nucleus of male mice. The changes in levels of these receptors may serve to balance the stress response. One theory is that CRFR1s are associated with an increased endocrine stress-response, while CRFR2s are associated with deceleration of the stress response. Therefore, conclusions from Ventura-Silva et al. suggest stress decreases CRF-signaling sensitivity. Several studies provide evidence for this theory; CRFR1 antagonism in the BNST reliably blocks the behavioral effects of CRF infusion (Sahuque et al., 2006) and chronic (Hu et al., 2020a) and early life stress (Hu et al., 2020a,b), while CRFR2 antagonism does not block CRF-infusion induced avoidance behavior in the EPM (Sahuque et al., 2006). In fact, CRFR2-deficient mice demonstrate increased avoidance behavior (Bale et al., 2000). ICV infusion of a CRFR2 antagonist increases cFOS and CRF expression in fear-related circuitry (Skorzewska et al., 2011). Opposing evidence, however, indicates that CRFR2 activity may be aversive and increase avoidance behavior. When a CRFR2 antagonist is co-infused with CRF into the BNST, it blocks CRF-induced avoidance in the conditioned place preference test (Sahuque et al., 2006). ICV administration of a CRFR2 antagonist attenuates RS-induced avoidance in rats undergoing ethanol withdrawal (Valdez et al., 2003). CRFR2 activity in the amygdala and hypothalamus activates the HPA axis under basal conditions and heightens RS-induced avoidance (Jamieson et al., 2006). Therefore, further investigation is needed to understand the differential roles of CRF receptors and their interactions with stress.

A compensatory negative feedback system may further complicate the interactions between CRF, CRFRs, and stress, because CRFRs are prone to upreglation and downregulation, dependent on CRF concentrations. CRF knockout in VTA-projecting neurons increases CRFR1 in the VTA of male mice (Dedic et al., 2018). In the BNST, overexpression of CRF decreases CRFR1 levels (Sink et al., 2013b) and CRFR1 mRNA expression (Regev et al., 2011). CRF-binding in BNST may induce both internalization and downregulation of CRFR1 (Perry et al., 2005). Experimental questions about how stress alters CRFRs should consider how stress indirectly changes CRFR expression via fluctuations in CRF. Importantly, all of these described experiments used male rodents. As discussed in later sections, CRF receptor expression differs between sexes, so further investigation is required to understand how sex differences affect CRF signaling.

Binding at either CRFR1 or CRFR2 triggers signal transduction pathways resulting in increased protein kinase A (PKA) production (Litvin et al., 1984; Chen et al., 1986; Battaglia et al., 1987). PKA-signaling pathways may lead to activation in the BNST through phosphorylation of glutamate receptors (Uematsu et al., 2015). Importantly, early life stress and CVMS paradigms result in increased CRF and PKA expression in the BNST (Hu et al., 2020a,b). PKA antagonism ameliorates the effects of CVMS on avoidance behaviors (Hu et al., 2020a). Taken together, evidence suggests that PKA is important for the regulation of stress-induced synaptic changes that produce long-lasting behavioral changes.

Another component of CRF signaling involves CRF-binding protein (CRF-BP), which is a membrane-associated protein proposed to bind to and dimerize CRF, clearing it from the bloodstream (Behan et al., 1995). The function of CRF-BP, and its interaction with stress, is not fully understood. CRF-BP-deficient mice exhibit increased baseline avoidance behavior and a slowed return to homeostasis after exposure to stress (Karolyi et al., 1999). Klampfl et al. (2016) found that inhibition of CRF-BP in the BNST impairs maternal care in lactating dams exposed to stress. This finding promotes a simple mechanism, where inhibition of CRF-BP increases free CRF. However, opposing evidence found by Vasconcelos et al. (2019) suggest the role of CRF-BP is not straightforward: CRF-BP antagonism restores social approach in male rats exposed to intermittent social defeat. This finding supports an alternate hypothesis that CRF-BP amplifies CRF signaling, perhaps by lengthening the half-life of CRF or promoting binding at CRFRs (Ketchesin and Seasholtz, 2015). Research on CRF-BP in the BNST is lacking, and future studies are required to determine whether these differences are due to dual mechanisms, compensatory mechanisms, sex differences, or another explanation. Future investigations are also required to determine whether stress affects the levels of CRF-BP in the BNST.

Stress also alters CRF-signaling via interactions with the upstream regulators PACAP and STEP. PACAP binds to its receptor PAC1 to act as an upstream regulator of the CRF signaling pathway. PACAP signaling is necessary and sufficient for the behavioral and endocrine stress-response and regulates avoidance behavior (Hammack et al., 2009; Kocho-Schellenberg et al., 2014). Importantly, CVMS increases PACAP and PAC1 in BNST of male rodents (Hammack et al., 2009; Hu et al., 2020a,b). Furthermore, PACAP signaling increases avoidance behavior and endogenous corticosterone levels while PAC1 antagonism reduces avoidance behavior (Roman et al., 2014). STEP acts as an upstream regulator by inhibiting CRF-signaling. CVMS decreases STEP levels in BNST (Hammack et al., 2009; Hu et al., 2020a,b) and chronic RS reduces STEP mRNA (Dabrowska et al., 2013) in anterolaterally located subnuclei. RS-induced decreases in STEP result in increased LTP in BNST neurons of male rats (Dabrowska et al., 2013), likely via attenuation of pathways that end in dephosphorylation of NMDARs (Olausson et al., 2012). Ultimately, stress increases CRF expression in the BNST by increasing upstream promotor PACAP and PAC1, while decreasing inhibitor STEP.

The described stress-induced changes to the CRF-signaling pathway are at least partially responsible for changes to BNST neuronal excitability. Overall, evidence indicates that stress increases excitability of BNST neurons. RS-induced neuroplasticity alters LTP firing rates in CRF+ BNST neurons (Dabrowska et al., 2013). Chronic unpredictable or variable stress in male rodents increases excitability in anterolateral and anterodorsolateral regions of the BNST, as measured by increased amplitude of miniature excitatory postsynaptic currents (mEPSC; Hu et al., 2020a,b) and evoked inhibitory postsynaptic currents (IPSCs; Partridge et al., 2016). Chronic RS also generates increased evoked excitatory postsynaptic currents (Dabrowska et al., 2013). Both chronic RS and CVMS cause alterations to the resting membrane potential (Snyder et al., 2019; Hu et al., 2020a,b), and decrease M-currents (Hu et al., 2020a,b), which help maintain membrane homeostasis via potassium channels. This stress-induced excitability can also be observed by increased expression of the neuronal activity marker cFOS expression in BNST (Ventura-Silva et al., 2012; Hu et al., 2020a,b). Taken together, these data demonstrate that stress exposure results in long-lasting changes in synaptic properties of BNST neurons through activating CRF signaling. CRF in the absence of stress (repeated ICV infusions) decreases LTP of intrinsic neuronal excitability, decreasing firing threshold and increasing temporal fidelity of firing, in the juxtacapsular BNST (Francesconi et al., 2009). Chronic unpredictable stress also increases connectivity between CRF+ neurons in the CeA and BNST (Partridge et al., 2016). Therefore, increased stress-induced excitability in CRF+ BNST neurons may also sensitize fear circuitry, driving increased avoidance and startle behaviors historically associated with anxiety.

In summary, chronic stress can result in long-lasting increases in BNST CRF expression, modify CRFR density in the BNST, and alter components of CRF-signaling to ultimately increase neuronal excitability. Increased activation of GABAergic CRF+ BNST neurons likely increases inhibition of other BNST projections to the hypothalamus, ultimately disinhibiting the HPA axis (Figure 2). These stress-induced synaptic changes to CRF+ neuron activity result in long-lasting behavioral changes in rodents.

### Implications for corticotropin-releasing factor bed nucleus of the stria terminalis in reward processing

In addition to regulating avoidance behaviors, neuroplastic changes to CRF + BNST neurons may also have implications for reward-related behaviors. Stimulation of adBNST projections to the VTA (Dong and Swanson, 2004a) is rewarding, and promotes place-preference (Kim et al., 2013). Some evidence indicates that CRF is implicated in the valence surveillance role of the BNST, by both promoting avoidance and regulating reward circuitry. CRF+ BNST neurons synapse at dopaminergic VTA neurons, which express both CRFR1s (Dedic et al., 2018) and CRFR2s (Rinker et al., 2017). CRF released from the BNST plays a neuromodulatory role in the VTA, inducing bimodal electrophysiological responses in VTA neurons (Wanat et al., 2013; Williams et al., 2014). More specifically, CRFR1-binding in VTA drives an increase in EPSCs at low CRF concentrations and CRFR2-binding drives attenuation of EPSCs and potentiation of IPSCs at high CRF concentration (Williams et al., 2014). Behaviorally, CRF infusion to the VTA reduces motivation in a progressive ratio task (Wanat et al., 2013) and reduces reward-evoked dopamine release from the nucleus accumbens. However, simultaneously stimulating the BNST reverses this effect on dopamine release (Wanat et al., 2013).

CRF is also implicated in multiple processes that drive drug and alcohol abuse: negative valence avoidance behavior during withdrawal, and positive valence reward-processing behavior during drug approach and drug use. CRF+ BNST inputs to the VTA at CRF1Rs are necessary for binge-like ethanol consumption in a mouse model of ethanol dependence (Rinker et al., 2017). Interestingly, some evidence indicates that stress exposure in rodents results in neuroplastic changes similar to those found in rodent models of drug and alcohol abuse, as reviewed by Cui et al. (2013) and Harris and Winder (2018). Like chronic stress, ethanol withdrawal increases CRF in the BNST (Olive et al., 2002). CRFR2 binding typically reduces EPSCs and amplifies IPSCs, which are diminished in rat VTA neurons after chronic cocaine self-administration (Williams et al., 2014). Both chronic stress and alcohol exposure disrupt norepinephrine signaling at CRF neurons in the BNST (Snyder et al., 2019). Changes at the synaptic level, such as reduced activity in CRF+ VTA-projecting BNST neurons (Silberman et al., 2013) may also lead to long-lasting mood-related symptoms of withdrawal known to drive excessive alcohol intake.

### Bed nucleus of the stria terminalis neuroplasticity in humans

Direct evidence of neuroplasticity in the human BNST is lacking, due to difficulties using non-invasive techniques to measure synaptic changes in deep brain regions. Diffusion Tensor Imaging (DTI) and functional Magnetic Resonance Imaging (fMRI) can be used to measure activity and functional connectivity of the BNST (Avery et al., 2014). Some studies measure differences in brain activity at baseline versus during exposure to fearful stimuli. Similar to what is observed in rodents, threat exposure elicits BNST activity in humans (Alvarez et al., 2011; Choi et al., 2012; McMenamin et al., 2014; Klumpers et al., 2015).

Other studies comparing subjects with stress-related mood disorders to healthy control subjects demonstrate that BNST is associated with maladaptive stress in humans. Humans with diagnosed anxiety disorders (Buff et al., 2016, 2017; Brinkmann et al., 2017) or high anxiety scores (Brinkmann et al., 2018) exhibit amplified BNST activity when exposed to unpredictable threat. Unpredictable threat cues also elicit greater BNST response in subjects with high social anxiety scores and in veterans with PTSD, when compared to less anxious controls. When shown unpredictably neutral or threatening images, subjects with PTSD or social anxiety disorder show greater connectivity between the BNST and the hippocampus, amygdala, insula (Feola et al., 2021b), ventromedial prefrontal cortex (Clauss et al., 2019; Feola et al., 2021b) and cingulate cortex (Clauss et al., 2019), compared to control subjects. Altered BNST connectivity is also associated with other psychiatric disorders, including schizophrenia (Feola et al., 2021a) and alcohol use disorder (Flook et al., 2021).

Finally, increased functional connectivity is found in caudate and striatum areas that are highly associated with the BNST upon threat exposure; however, spatial resolution limitations make it unclear whether these changes are occurring in BNST (McMenamin and Pessoa, 2015). Bi-directional changes in efficacy in functional connections may indicate neuroplasticity at these regions in humans, though further investigation is required to determine whether changes are localized at the BNST.

## Sex differences in corticotropin-releasing factor signaling in the bed nucleus of the stria terminalis

The BNST in male and female rodents is anatomically diverse, varying in size, receptor affinity and expression, cell type, and interconnectivity. Sex differences in synaptic properties of BNST neurons may help explain behavioral differences after stress exposure in male and female rodents.

Rodent studies have demonstrated gonadal hormones can promote divergence in BNST size. Although the total BNST volume does not differ between sexes, several subdivisions show bidirectional sexual dimorphism in volume and neuron number. Suppression of gonadal hormones during development, via orchiectomy or androgynization shortly after birth, decreases sex-dependent size differences in medial anterior and medial posterior regions of the rat BNST which are typically smaller and larger, respectively, in males (del Abril et al., 1987; Guillamon et al., 1988; Segovia and Guillamon, 1993). Neuron number is also greater in the anterolateral region of female rats and altering gonadal hormone levels through androgynization results in a decrease in neuron numbers to below those of control males (Guillamon et al., 1988). Gonadal hormones not only indirectly influence BNST size but play a direct role in BNST action. In the BNST of male rats, estrogen receptor (ER) α, (Wu et al., 2009), aromatase (Tabatadze et al., 2014) and androgen receptors (Herbison, 1995; Brock et al., 2015) are more densely expressed relative to female rats. Testosterone action is not only limited by decreased AR expression, but cytochrome P-450 aromatase in females is less efficient at converting testosterone to estrogen (Roselli et al., 1996).

Disparities in receptor affinity in CRF + BNST neurons help to further explain differences in sensitivity to stress and stress hormones. The Gsα subunit of G-proteins couples more effectively with CRFR1 in females, compared to males, causing a larger neuronal response (Bangasser et al., 2010). Conversely, CRF binding at CRFR2 is greater in some subnuclei in males, which may have stress-suppressive effects (Weathington et al., 2014). Receptor affinity may explain greater sensitivity to stress, and more intense and persistent HPA-axis activation in females (Critchlow et al., 1963; Kant et al., 1983; Williams et al., 1985; Brett et al., 1986). This hypothesis is complicated by evidence that stress-induced corticosteroid release is greater in male rats, relative to females (Karandrea et al., 2000, 2002). Other components of the CRF signaling pathway differ between sexes, including whole-brain differences that likely affect the dense population of CRF neurons in the BNST. Female rats express higher levels of PAC1 mRNA compared to males. PAC1R antagonism affects female, not male, cued fear learning (Kirry et al., 2018), and plays a significant role in stress-induced plasticity (Hammack et al., 2009; Hu et al., 2020a,b). Sex-specific sensitivity in PACAP signaling is also evident in humans, wherein female, compared to male, PTSD patients have a stronger correlation between the risk and severity of PTSD and the single nucleotide polypeptide (SNP) for CRFR2 and the SNP for PACAP (Ressler et al., 2011).

The human BNST also exhibits differences based on sex as defined by the type of gonads present, a phenomenon that may explain why cisgender women are twice as likely to develop mood disorders, including PTSD (Breslau, 2001; Kessler et al., 2003), a mood disorder highly associated with the BNST (Lebow and Chen, 2016). The BNST in men and women differs in size. One subnucleus of the human BNST, the central nucleus, is larger in males and contains twice as many somatostatin neurons relative to females. Most interestingly, transgender women (assigned male at birth) have a female-typical, smaller central nucleus with fewer somatostatin neurons, and vice versa (Zhou et al., 1995; Kruijver et al., 2000; Pol et al., 2006; Swaab, 2007). However, these studies cannot remove the confounding factor of elevated chronic psychosocial stressors experienced by trans individuals, which would influence BNST connectivity and neuroplasticity. There also appear to be sex differences in functional connectivity; DTI and fMRI indicate that 76% of brain structures have greater BNST structural connectivity in females, rather than males (Avery et al., 2014). Sex differences are evident in the BNST and in key components of stress-induced neuroplasticity, including CRF, PACAP, and their associated receptors. Sex differences at baseline may result in differing synaptic sensitivity to stress. Further investigation is required to understand how stress differentially alters the synapse of BNST CRF+ neurons in males and females.

## Summary

Exposure to chronic stress results in long-lasting changes to synaptic properties of CRF-expressing neurons in the BNST. This neuroplasticity is associated with behavioral changes in rodents, including increased avoidance of aversive contexts. These behaviors are historically associated with anxiety and are sensitive to anxiolytic drug interventions. The cross-species applications of this mechanism are evident, as stress and anxiety are also associated with the human BNST and CRF-signaling pathways. Research on stress-induced neuroplasticity is timely, especially as the current worldwide COVID-19 pandemic, and the resulting economic, financial, social and healthcare troubles, have increased chronic stress-related mood disorders such as anxiety and depression (Lee et al., 2020; Salari et al., 2020). This increase further intensifies the already urgent need for research on the neural circuitry responsible for chronic stress-related maladaptation (Binder and Nemeroff, 2010). Currently, there is evidence that deep brain stimulation of the BNST ameliorates stress-related mood disorders, including obsessive compulsive disorder (Winter et al., 2018), anorexia nervosa, and major depressive disorder (Blomstedt et al., 2017). Therapeutic approaches that more specifically disrupt stress-induced changes to CRF signaling in the BNST could prevent or ameliorate the long-term effects of chronic stress exposure (Sanders and Nemeroff, 2016).

## Author contributions

IM performed the literature review and wrote the first draft of the manuscript. BS and TR provided revisions. All authors contributed to the article and approved the submitted version.
